# Reciprocal Associations Between Eating Pathology and Parent-Daughter Relationships Across Adolescence: A Monozygotic Twin Differences Study

**DOI:** 10.3389/fpsyg.2018.00914

**Published:** 2018-06-07

**Authors:** Laurel M. Korotana, Kristin M. von Ranson, Sylia Wilson, William G. Iacono

**Affiliations:** ^1^Department of Psychology, University of Calgary, Calgary, AB, Canada; ^2^Department of Psychology, University of Minnesota, Minneapolis, MN, United States

**Keywords:** eating disorders, disordered eating, parent-child relationship, non-shared environment, monozygotic twins, family, MZ twin differences

## Abstract

This prospective study explored longitudinal, bidirectional associations between eating pathology and perceptions of the parent-child relationship (i.e., parent-child regard and involvement) across adolescence. Specifically, this study examined whether twin differences in mother-daughter and father-daughter relationship problems emerged as a risk factor for, or outcome of, twin differences in eating pathology. By examining twin differences, this study explored associations between variables while controlling for shared environmental and genetic effects. A population-based sample of 446 monozygotic twin girls and their mothers completed questionnaires when twins were approximately 11, 14, and 17 years. Responses were analyzed using longitudinal cross-lagged models. Overall, few strong longitudinal associations were observed. Where longitudinal associations emerged, overall patterns indicated reciprocal associations that shifted across adolescence. Whereas twin differences in parent-daughter relationship variables more often predicted later twin differences in eating pathology across early adolescence, conversely, twin differences in eating pathology more often predicted later twin differences in parent-daughter relationship variables across later adolescence. In particular, the twin who reported greater eating pathology later reported more negative perceptions of the father-daughter relationship, as compared to her co-twin. Findings raise questions for future research regarding parental—in particular, paternal—responses to adolescent eating pathology and suggest the potential importance of efforts to support the parent-daughter relationship within the context of adolescent eating pathology.

## Introduction

Eating disorders are associated with severe negative implications for social, emotional, and functional well-being, and are often accompanied by serious medical complications (Klump et al., 2009). Based on DSM-5 criteria for eating disorders, approximately 13% of females may develop clinically significant eating pathology by young adulthood (Stice et al., 2012). In addition, subclinical eating pathology—which is often associated with negative psychosocial and medical challenges similar to those observed among clinical eating disorders (Thomas et al., 2009)—is even more prevalent. Among adolescent girls, research suggests 46–80% are dissatisfied with their weight, 26–77% have dieted, and 5–16% have engaged in compensatory behavior (e.g., self-induced vomiting or use of laxatives or diuretics; Chamay-Weber et al., 2005). Recognizing the widespread prevalence of eating pathology (i.e., both clinical and subclinical symptoms of eating disorders) and substantial implications for health and psychosocial well-being, understanding and ultimately reducing eating pathology is imperative.

It is widely accepted that eating pathology is a multiply-determined problem involving complex interactions among genetic and environmental factors (e.g., Striegel-Moore and Bulik, 2007). One factor that has oft been considered in the development of eating pathology is the parent-child relationship. A better understanding of the specific nature of associations between eating pathology and the parent-child relationship may have implications for eating pathology prevention and treatment research, including development of programs to reduce eating pathology.

### Eating pathology and the parent-child relationship

Although now controversial, families—in particular, parents—were historically theorized to be a primary cause of the development of eating pathology in children and adolescents (e.g., Gull, 1874). However, perspectives regarding the role of parents in child and adolescent eating pathology began to shift around the 1960s. For example, Minuchin et al. (1978) emphasized that parents should not be blamed for adolescent eating pathology, yet continued to theorize that certain pathological interactive familial processes (e.g., enmeshment, rigidity, conflict avoidance) were fundamental to the pathogenesis of eating pathology, particularly anorexia nervosa (AN).

This view has been supported by research demonstrating parent-child relationship problems and general family dysfunction are associated with eating pathology in children and adolescents. For example, multiple large-scale reviews that have examined putative risk factors for eating disorders (e.g., Jacobi et al., 2004; Holtom-Viesel and Allan, 2013) indicated various aspects of parent-child relationships and family environment are more dysfunctional among individuals with eating disorders than individuals without eating disorders. Parental and family factors suggested to be associated with eating pathology have included low parent-child connectedness and communication (e.g., Berge et al., 2014); insecure parent-child attachment (e.g., Goossens et al., 2012); low parent-child regard (e.g., Ackard et al., 2006); parental overprotection and high control (e.g., Salafia et al., 2009); low parental monitoring (e.g., Krug et al., 2014, 2016); problematic parental bonding (Tetley et al., 2014); and family weight-, food-, and appearance-related teasing (e.g., Eisenberg et al., 2012). However, there is little evidence of a consistent pattern of family dysfunction.

### Methodological limitations

Methodological shortcomings of research that explores eating pathology and parent-child relationships significantly limit conclusions that may be drawn. For example, the majority of research in this area has relied on cross-sectional designs. Cross-sectional research can identify correlates of eating pathology but cannot differentiate variables as risk factors, concomitants, or consequences of eating pathology. Establishing correlates of an outcome is an important first step in identifying risk factors. Furthermore, establishing the temporal precedence of a proposed risk factor to a proposed outcome is a crucial criterion for distinguishing risk factors from correlates, which typically requires a longitudinal design (Kraemer et al., 1997). In addition, research often has used only a single informant (typically, the child or adolescent) and exclusively considered the mother-child relationship while overlooking the father-child relationship. Most research has not been genetically informed, failing to account for the moderate to large heritability of eating pathology (Thornton et al., 2011). It is necessary to account for genetic effects to disentangle confounded genetic and environmental sources of variance in eating pathology, and rule out possibilities such as (a) a genetically-transmitted liability explains both parent-child relationship variables and eating pathology, (b) a genetically-influenced disposition evokes certain parenting responses and thereby impacts the parent-child relationship, or both. Research often has failed to differentiate overall family climate (a shared environmental factor) from issues specific to the parent-child relationship (a non-shared environmental factor), despite research consistently indicating non-shared environmental factors are responsible for most environmental variation relevant to psychological development (Plomin, 2011). More specifically, research has suggested non-shared environmental factors account for 44–54% of the variance in adolescent eating pathology (Klump et al., 2007). Finally, research tends to have lacked a developmentally appropriate perspective. Beginning assessment at or before pre-adolescence, extending assessment through adolescence, and clearly defining the specific age span examined are important, so as to identify associations between variables that may shift across developmental periods.

### Conceptual shift: risk factor or outcome?

There has been a recent conceptual shift regarding the role of the parent-child relationship in child and adolescent eating pathology, which provides the impetus for the current study. Rather than emphasizing parental etiological factors in the development of eating pathology, researchers have increasingly emphasized parental maintenance mechanisms in the progression of eating pathology. Further, many researchers have hypothesized that parent-child relationship difficulties are likely outcomes, rather than causes, of living with a child with a condition such as eating pathology that threatens medical and mental health (Sim et al., 2009; Nilsson et al., 2012).

Unfortunately, most studies tend to have examined parent-child relationship variables as predictors of the later development of eating pathology without considering the reverse possibility—i.e., that eating pathology may predict later parent-child relationship problems. However, some exceptions exist. Strong empirical support for the perspective that eating pathology precedes parent-child relationship problems was provided by a longitudinal, population-based study by Spanos et al. (2010). This study examined associations between eating pathology and parent-child conflict across adolescence among a sample of female twins. A rigorous analytic approach was used that considered bidirectional associations between variables (i.e., parent-child conflict could predict eating pathology and/or eating pathology could predict parent-child conflict). Findings indicated parent-child conflict was not a precipitant of eating pathology; rather, parent-child conflict at age 17 years was a consequence of eating pathology (in particular, weight preoccupation) at age 14 years. The current study used the same methodology and sample as Spanos et al. (2010) (see below). Longitudinal treatment research has also indicated improvements in family relationships (e.g., increased closeness, improved communication) are associated with recovery from adolescent-onset eating disorders (Nilsson et al., 2012), regardless of treatment type (Ciao et al., 2015).

Other research that has considered bidirectional associations between eating pathology and parent-child relationship variables provides somewhat conflicting results. For example, adolescent girls' body satisfaction has been demonstrated to be both affected by and a predictor of perceived family connectedness (Crespo et al., 2010). Further, research with a community sample of adolescent girls indicated lower parent-child relationship quality at age 12 years predicted greater dieting at age 13 years, and not the reverse (Archibald et al., 1999), although greater dieting and food preoccupation at age 14 years predicted lower parent-child relationship quality at age 16 years, and not the reverse (Archibald et al., 2002). Possibly, parent-child relationship problems are both risk factors for *and* outcomes of eating pathology, with the nature of associations shifting depending on specific variables assessed and the developmental period examined.

### Purpose

Researchers are calling for more methodologically rigorous research regarding eating pathology and parent-child relationships to better understand associations between variables (e.g., Le Grange et al., 2010). The overarching goal of this study was to address this call. *Specifically, the purpose of the current study was to examine longitudinal, bidirectional associations between eating pathology and perceptions of the parent-daughter relationship across adolescence using a methodologically rigorous research design that overcame several methodological challenges that have limited previous study conclusions*. This study extended previous work by Spanos et al. (2010), reviewed above, that examined associations between eating pathology and parent-child conflict. Similar sample and methodology were used: (1) a monozygotic (MZ) twin differences design that controlled for confounding genetic and shared environmental factors among MZ twins (see Data Analyses section for details); (2) cross-lagged models were run that considered longitudinal, bidirectional associations among variables; and (3) a developmental perspective was incorporated that included and differentiated early and late adolescent periods. The current study extended analyses beyond those included by Spanos et al. by:

1. Separately examining the mother-daughter and father-daughter relationship;
2. Exploring whether the strength of longitudinal associations observed in the full sample differed when examined in the twin pairs most discordant for eating pathology, as some previous research that has examined twin differences in parent-child relationship variables and adolescent psychosocial outcomes has indicated more robust associations between variables among twin pairs most discordant on psychosocial outcomes (Asbury et al., 2003; Burt et al., 2006);
3. Considering both the daughter and mother's perspectives of the parent-daughter relationship; and
4. Examining the parenting constructs of parent-child involvement and regard rather than parent-child conflict. As noted above, parent-child involvement and regard have been associated with eating pathology in previous research (e.g., Ackard et al., 2006; Berge et al., 2014; Krug et al., 2014), although conclusions have been limited by methodological issues. As such, it remains uncertain whether low parent-child involvement and regard are more likely to contribute to, or result from, eating pathology. Understanding the nature of associations between these variables is important for improving understanding of the development and maintenance of eating pathology, best supporting parent-child relationships during periods of risk for eating pathology, and navigating how to best include parents in eating disorder treatment programs. Notably, both parent-child involvement and parent-child regard are individual aspects of parenting behaviors that represent modifiable parenting practices, rather than more “trait-like” parenting styles. Thus, they are particularly important to study, as they represent variables that can be feasibly targeted for change to promote improved adolescent outcomes.

We hypothesized that twins with poorer parent involvement and regard would report more subsequent eating pathology than their co-twin.

## Materials and methods

### Participants

Participants included 446 female MZ twins (i.e., 223 twin pairs) and their mothers [i.e., 204 mothers, 99.5% (*n* = 203) biological mothers] who participated in the Minnesota Twin Family Study (MTFS). The MTFS is an ongoing population-based, longitudinal study that originated in 1991 and involves assessment at 3-year intervals of same-sex twins from Minnesota and their parents (Iacono and McGue, 2002). Families were excluded from participation if the twins were adopted; did not live within a day's drive to the University of Minnesota's Twin Cities campus; or if one or more twins was deceased or had a mental or physical handicap that would impede completion of assessments. Twins were identified through birth records with 90% of all twins born in Minnesota between 1971 and 1985 successfully contacted. Of those contacted, 83% agreed to participate. Only data from MZ twins were used in the current study to facilitate an MZ twin differences design that specifies non-shared environmental effects.

At intake, there were no significant differences in rates of parental psychopathology or self-reported socioeconomic status between MTFS participants and other families in Minnesota with adolescent twins (Iacono et al., 1999). In addition, the ethnic composition of the MTFS sample was representative of the ethnic composition of Minnesota at the time of recruitment (over 95% Caucasian).

Participants were assessed at pre-adolescence (i.e., age 11 years), middle adolescence (i.e., age 14 years), and late adolescence (i.e., age 17 years). Beginning assessment at pre-adolescence and extending assessment through adolescence is important, as adolescence and the numerous changes involved in this developmental period—e.g., puberty, physical maturation, development of greater self-identity, shifting importance of familial vs. peer relationships—have been associated with high risk for the development of eating pathology (von Ranson and Wallace, 2014).

Data provided by twins' fathers were not included as questionnaires relevant to the current study were not administered to fathers at all three time points.

### Attrition and missing data

Of the 446 MZ twins included in the current study, self-report data were available for 86% and 79% of twins at age 14 and 17 years, respectively. Maternal reports of the mother-child relationship were available for 90% of twins at age 11 years, 82% of twins at age 14 years, and 74% of twins at age 17 years. Some measures were inadvertently not administered to some participants at specific time points. In addition, some participants were missing responses for specific scale items. By convention, total scale and subscale scores were prorated if no more than 10% of items were missing.

To explore any effect of attrition and missing data on findings, preliminary analyses used independent sample *t*-tests to compare variables of interest (i.e., eating pathology and parent-child relationship) at each time point between participants who had available data on the variable of interest at all three time points vs. only one or two time points. Results indicated almost no significant differences between groups on any variable measured at any time point, with a few exceptions: twin ratings of maternal regard for the twin at age 11 years were significantly lower in the group with complete data, and twin ratings of their regard for their father at age 14 years and binge eating at age 14 years were significantly higher in the group with complete data.

### Measures

Internal consistency reliabilities for all measures across time-points are listed in Table 1.

**Table 1:** Internal consistency reliability coefficients (α) for all measures administered across time points and raters.

	**Age 11**	**Age 14**	**Age 17**
**TWIN RATINGS**			
MEBS Total	0.86	0.94	0.93
MEBS Body Dissatisfaction	0.82	0.90	0.90
MEBS Weight Preoccupation	0.79	0.90	0.89
MEBS Binge Eating	0.61	0.81	0.81
PEQ Mother Regard for Daughter	0.58	0.66	0.78
PEQ Father Regard for Daughter	0.58	0.79	0.81
PEQ Daughter Regard for Mother	0.58	0.84	0.84
PEQ Daughter Regard for Father	0.79	0.88	0.90
PEQ Mother Involvement	0.71	0.80	0.86
PEQ Father Involvement	0.79	0.85	0.91
**PARENT RATINGS**			
PEQ Mother Regard for Daughter	0.40	0.62	0.55
PEQ Daughter Regard for Mother	0.79	0.80	0.77
PEQ Mother Involvement	0.75	0.79	0.83

*MEBS, Minnesota Eating Behaviors Survey; PEQ, Parental Environment Questionnaire*.

#### Eating pathology

Eating pathology was assessed using the Minnesota Eating Behavior Survey^1^ (MEBS; von Ranson et al., 2005), a 30-item self-report questionnaire assessing current disordered eating attitudes and behaviors. The MEBS can be reliably administered to children as young as 10 years of age.

The MEBS provides a Total Score and four subscale scores: Body Dissatisfaction (i.e., discontent with body size and shape—e.g., “My stomach is too big;” 6 items), Weight Preoccupation [i.e., preoccupation with weight, eating, and dieting—e.g., “I think a lot about dieting (or losing weight)”; 8 items], Binge Eating (i.e., binge eating, secretive eating, and preoccupation with food—e.g., “I eat when I'm upset about things;” 7 items), and Compensatory Behavior (i.e., use of, and thoughts of using, self-induced vomiting and other inappropriate compensatory behaviors to control weight—e.g., “I have thought about throwing up (vomiting) to lose weight;” 6 items). As such, the types of eating pathology examined in the current study included both cognitive and behavioral symptoms of eating disorders.

Previous research with the larger MTFS sample indicates satisfactory internal consistency for the MEBS Total scale and Body Dissatisfaction, Weight Preoccupation, and Binge Eating subscales (von Ranson et al., 2005). In contrast, the Compensatory Behavior subscale has demonstrated low internal consistency (e.g., alpha = 0.40) and infrequent item endorsement in early adolescents (e.g., von Ranson et al., 2005). Thus, this subscale was excluded from analyses in the current study. Three-year test-retest reliability of the MEBS is modest (von Ranson et al., 2005).

At age 11 years, the version of the MEBS administered included a two-point “true or false” response scale. At age 14 and 17 years, the response format was expanded to a four-point scale (*Definitely True, Probably True, Probably False, Definitely False*). To maintain consistency across assessment points, the current study combined *Definitely True* and *Probably True* responses, and *Definitely False* and *Probably False* responses, at age 14 and 17 years.

#### Parent-daughter relationship

Perceptions of the parent-daughter relationship were measured via the Parental Environment Questionnaire (PEQ; Elkins et al., 1997), a 50-item self-report questionnaire that can be reliably administered to children as young as 10 years of age. There are numerous informant variants: e.g., each twin reports on her relationship with each parent separately and each parent reports on her relationship with each twin separately. As some families included biological parents and step-parents, twins rated the mother and father they lived with the most and the same parent provided ratings of the twin. If a biological parent was deceased or otherwise unavailable (e.g., had lost contact with the twin), ratings for and from that parent were indicated as missing. Items were essentially the same across informant versions, except wording was changed to suit the particular rater and parent-child relationship being rated. Each item is rated on a four-point Likert scale (*Definitely True, Probably True, Probably False, Definitely False*).

The PEQ is composed of five scales: Conflict (i.e., extent to which the parent-child relationship is characterized by disagreement, tension, and anger—e.g., “This child and I often get into arguments;” 12 items), Involvement (i.e., extent to which the parent-child relationship is characterized by communication, closeness, and support—e.g., “This child talks about his/her concerns and experiences with me;” 12 items), Regard for Parent (i.e., extent to which the child is proud of and respects the parent—e.g., “This child wants to be like me in a number of ways;” 8 items), Regard for Daughter (i.e., extent to which the parent has high regard for the child—e.g., “I love this child no matter what he/she does;” 5 items), and Structure (i.e., extent to which the parent sets rules, monitors compliance, and believes compliance is important—e.g., “I make it clear what I want this child to do or not do;” 5 items). Only the Involvement, Regard for Parent, and Regard for Daughter scales were used in the current study. Higher scale scores indicate greater involvement, regard for parent, and regard for child, respectively. These three scales have demonstrated adequate internal consistency across parent and child ratings (e.g., alpha = 0.62–87; Elkins et al., 1997). The Conflict scale was not used, as it has already been examined in a similar study by Spanos et al. (2010; see above). The Structure scale was not used, as previous research has indicated low internal consistency of this scale across age and informant ratings (i.e., alpha = 0.46–0.58; Elkins et al., 1997).

#### Body mass index

Body mass index (BMI; i.e., one's body weight, measured in kilograms, divided by the square of one's height, measured in meters) of each daughter was calculated from weight and height measurements taken by research staff using a level platform physician's scale. Specifically, linear regression analyses were used to regress out each twin's BMI from their MEBS scores at all ages. Standardized residuals were then retained as an indicator of eating pathology that was not accounted for by BMI. Revised MEBS twin difference scores were then calculated by subtracting Twin B's revised MEBS score from Twin A's revised MEBS score at all ages.

### Data analyses

Cross-lagged analyses were performed using a structural equation modeling program, Mplus version 7.2 (Muthen and Muthen, 2010); all other analyses were performed using SPSS version 22 (IBM Corp, 2013).

Within-pair twin difference scores were used in all cross-lagged analyses, creating an MZ twin differences design. This design has been described as the most powerful and direct approach for identifying non-shared environmental influences on development (Mullineaux et al., 2009; Vitaro et al., 2009). By examining within-twin pair differences in non-shared environmental factors (i.e., parenting constructs, in the current study) and connecting these differences to within-twin pair differences in developmental outcomes (i.e., eating pathology, in the current study), associations can be identified between non-shared environmental factors and developmental outcomes. As MZ twins share 100% of their genes as well as certain aspects of their environment, genetic and shared environmental factors cannot account for differences in developmental outcomes between MZ twins from the same pair. In other words, the influence of genetic and shared environmental factors on developmental outcomes is the same for both twins, thus is methodologically controlled in the MZ twin differences design. As such, the association between difference scores reflects the contribution of non-shared experiences (i.e., parenting) to the creation of twin outcome differences (i.e., eating pathology), independent of either genetics or factors that differ between families.

To calculate twin difference scores, Twin B's score was subtracted from Twin A's score on each variable of interest. The sign (i.e., positive or negative) of scores was retained to establish the direction of differences within twin pairs.

To interpret correlation effect sizes, we used Cohen's (1992) proposed operational definitions of small (0.10), medium (0.30), and large (0.50) effects.

#### Full sample

Descriptive statistics for MEBS and twin-rated PEQ scores were computed, using individual twin scores and twin difference scores. Exploratory Pearson's correlations were run using individual twin scores to examine within-individual cross-sectional and longitudinal associations between MEBS and twin-rated PEQ scores. As the assumption of independent cases was violated (i.e., twin pair data are clustered due to familial-genetic characteristics), only one twin from each pair was randomly selected to be included in each correlation. Pearson's correlations were repeated with twin difference scores replacing individual twin scores, to verify whether any associations between MEBS and twin-rated PEQ scores remained when the parent-child relationship was isolated as a non-shared environmental factor.

**Figure 1:**
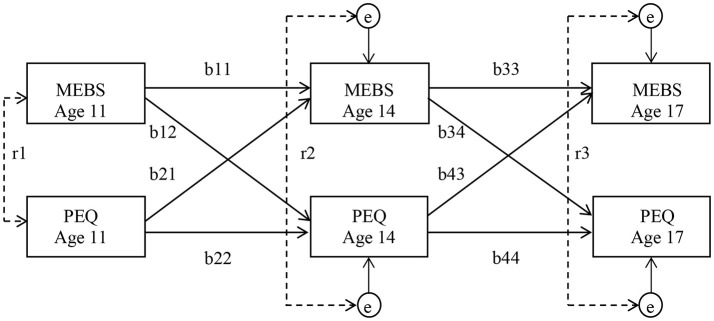
Structure of cross-lagged models examining longitudinal associations between eating pathology and the parent-child relationship. Partial regression coefficients are on the cross-age paths (b). Within-age correlations are illustrated by dotted lines (r). Due to the inclusion of stability and cross-lagged coefficients in the model, these within-age correlations function as residuals at ages 14 and 17 (e). Residuals reflect associations between MEBS and PEQ difference scores that are specific to the age at which they were measured and independent of any preexisting associations. Percentage of variance accounted for by each path can be obtained by squaring the path coefficient. MEBS, Minnesota Eating Behaviors Survey; PEQ, Parental Environment Questionnaire.

Following methods implemented by Burt et al. (2006), cross-lagged models were fitted to the twin difference scores to examine prospective, reciprocal associations between eating pathology and twin ratings of the parent-daughter relationship (see Figure 1 for the model's structure). Separate cross-lagged models were performed to analyze associations among each PEQ scale and each MEBS scale and subscale. Cross-lagged models included each variable at each time point, with cross-lagged paths that indicated the association between one variable at an earlier time point and another variable at a later time point. Regression paths between the same variable across adjacent time points reflect the relative temporal/positional stability of constructs across time. This design facilitated examination of directional and reciprocal associations. In the current study, this means two possibilities—(1) that twin differences in the parent-daughter relationship prospectively predicted twin differences in eating pathology, and (2) that twin differences in eating pathology prospectively predicted twin differences in the parent-daughter relationship—were examined. By controlling for pre-existing and within-age associations, these models only examine associations between adjacent ages (i.e., age 11 and 14, and age 14 and 17) and provide a particularly stringent test of longitudinal associations across time.

In addition, to examine to what extent, if any, associations between twin ratings of the parent-daughter relationship and eating pathology were accounted for by BMI, cross-lagged models were re-run with BMI regressed out from eating pathology scores.

Mplus' MLR estimator was used in all cross-lagged analyses. This estimator provides maximum likelihood parameter estimates with standard errors and a chi-square test statistic that are robust to non-normality (Muthen and Muthen, 2010). Adequate model fit was determined by a non-significant chi-square value, root mean square error of approximation (RMSEA) value of 0.05 or less, and comparative fit index (CFI) value of 0.90 or greater (Hu and Bentler, 1999; Kline, 2011; Byrne, 2012). Full information maximum likelihood (FIML) was used to accommodate missing data in cross-lagged models. A minimum of 10 cases to every parameter has been recommended to establish adequate power in path models (e.g., Kline, 2011). As such, sample size was considered adequate in cross-lagged analyses of the full sample.

#### Discordant subsamples

Cross-lagged analyses described above were re-run using only the subsamples of twin pairs with greatest discordance on eating pathology, to determine whether the strength of associations in the full sample differed among twin pairs most discordant for eating pathology. To select these subsamples, mean discordance for each twin pair across the three time points was calculated for each MEBS scale and a median split was performed. Only twin pairs that scored at or above the median for discordance on each MEBS scale were included in analyses of that scale.

Each discordant subsample included approximately half of the total sample (e.g., 111 twin pairs). Based on the number of parameters in the models (i.e., 17), the size of these discordant subsamples was smaller than recommended for adequate statistical power (Kline, 2011). However, these analyses are included because sample size was similar to that of discordant samples used in cross-lagged analyses that identified significant effects in previous research and use of discordant twin pairs is considered the most powerful means of identifying non-shared environmental effects (e.g., Burt et al., 2006).

#### Maternal perceptions of the mother-daughter relationship

Associations between maternal ratings of the mother-daughter relationship and eating pathology were examined in the full sample by re-running descriptive, correlational, and cross-lagged analyses (described above) with maternal ratings replacing twin ratings of the mother-daughter relationship.

#### Statistical significance

As numerous statistical analyses were included in the current study, probability of type I error was elevated. However, alpha level was retained at *p* < 0.05 for several reasons, including: (1) due to the exploratory nature of this research, it was considered beneficial to be less conservative in interpreting results to avoid overlooking any possible associations; (2) cross-lagged models analyzed in this research were already statistically rigorous (e.g., used twin difference scores, controlled for both stability and within-age associations, etc.); and (3) use of an alpha level of 0.05 is consistent with other studies that have performed cross-lagged models with twin difference scores (e.g., Burt et al., 2006; Spanos et al., 2010).

## Results

### Eating pathology and twin perceptions of the parent-daughter relationship

#### Descriptive statistics

Descriptive statistics for MEBS and twin-rated PEQ scores are presented in Table 2. A range of severity of eating pathology was observed at each time point. Comparison of MEBS data to clinical cutoffs for eating pathology identified by von Ranson et al. (2005) suggested a portion of the sample likely demonstrated clinical levels of eating problems. Specifically, 4% of twins scored at or above the clinical cutoff on the MEBS Total scale at age 11 years and 8% of twins met this cutoff at both ages 14 and 17 years.

**Table 2:** Descriptive statistics for eating pathology and twin ratings of the parent-child relationship.

**Variable**	**Age 11**					**Age 14**					**Age 17**				
	***M***	***SD***	**Min**.	**Max**.	***N***	***M***	***SD***	**Min**.	**Max**.	***N***	***M***	***SD***	**Min**.	**Max**.	***N***
**INDIVIDUAL TWIN SCORES**															
MEBS Total	5.43	4.73	0	21	414	5.63	5.64	0	23	385	6.20	5.59	0	24	352
MEBS Body Dissatisfaction	1.09	1.66	0	6	416	1.84	2.11	0	6	385	2.06	2.15	0	6	352
MEBS Weight Preoccupation	2.45	2.27	0	8	415	2.23	2.40	0	8	385	2.39	2.36	0	8	352
MEBS Binge Eating	1.05	1.32	0	7	415	0.84	1.30	0	7	385	1.00	1.35	0	7	352
PEQ Mother Regard for Daughter	19.30	1.24	14	20	440	18.84	1.67	11	20	342	18.68	2.09	7	20	373
PEQ Father Regard for Daughter	19.12	1.67	5	20	437	18.68	2.02	6	20	336	18.42	2.39	6	20	366
PEQ Daughter Regard for Mother	30.06	2.05	23	32	439	28.93	3.23	14	32	342	28.89	3.44	13	32	372
PEQ Daughter Regard for Father	29.38	3.08	8	32	435	28.06	4.00	8	32	335	27.78	4.54	8	32	365
PEQ Mother Involvement	42.18	4.52	26	48	440	40.48	5.33	22	48	342	40.41	5.93	19	48	372
PEQ Father Involvement	40.32	5.56	12	48	436	37.70	6.40	15	48	335	36.86	7.91	12	48	365
**TWIN DIFFERENCE SCORES**															
MEBS Total	3.40	2.94	0	16	198	3.76	3.85	0	19	183	3.87	4.12	0	24	162
MEBS Body Dissatisfaction	0.93	1.24	0	6	200	1.44	1.52	0	6	183	1.30	1.51	0	6	162
MEBS Weight Preoccupation	1.74	1.59	0	7	199	1.66	1.72	0	7	183	1.88	1.90	0	8	162
MEBS Binge Eating	1.02	1.15	0	7	199	0.91	1.18	0	5	183	0.94	1.13	0	6	162
PEQ Mother Regard for Daughter	0.97	1.29	0	6	217	1.17	1.46	0	7	166	1.45	1.91	0	11	176
PEQ Father Regard for Daughter	1.07	1.47	0	9	215	1.39	1.99	0	14	163	1.61	2.06	0	11	173
PEQ Daughter Regard for Mother	1.97	1.61	0	8	216	2.17	2.09	0	8	166	2.24	2.53	0	17	175
PEQ Daughter Regard for Father	2.14	2.16	0	14	213	2.79	3.18	0	22	162	2.58	2.79	0	17	172
PEQ Mother Involvement	4.12	3.44	0	15	217	3.67	3.12	0	16	166	4.21	3.82	0	22	175
PEQ Father Involvement	4.71	3.73	0	20	215	4.68	4.11	0	24	162	5.18	4.56	0	24	172

*The absolute values of twin difference scores are presented. MEBS, Minnesota Eating Behaviors Survey; PEQ, Parental Environment Questionnaire; min., minimum; max., maximum*.

#### Cross-sectional and longitudinal correlations

Within- and across-age correlations between MEBS and twin-rated PEQ scores are presented in Table 3, including correlations between individual twin scores (i.e., within-twin) and between twin difference scores (i.e., within-pair). Where statistically significant correlations were observed, associations were negative and effect sizes were small to medium. Individual twin correlations were generally stronger and more consistently observed within-age than across-age. Fewer significant correlations were observed when twin difference scores, rather than individual twin scores, were examined. As MZ twins share 100% of their genes, correlations between twin difference scores reflect non-shared environmental influences. Thus, the relative lack of significant correlations observed when we examined twin difference scores, rather than individual twin scores, suggests genetics and shared environment contribute most to associations between variables.

**Table 3:** (1) Within-twin Pearson correlations and (2) within-pair twin difference score Pearson correlations (displayed in parentheses) for eating pathology and twin ratings of the parent-child relationship (*N* = 223).

	**Age 11**				**Age 14**				**Age 17**			
	**Total**	**BD**	**WP**	**Binge**	**Total**	**BD**	**WP**	**Binge**	**Total**	**BD**	**WP**	**Binge**
**PEQ MOTHER REGARD FOR DAUGHTER**												
Age 11	−0.25^**^ (−0.15^*^)	−0.24^**^ (−0.20^**^)	−0.17^*^ (−0.09)	−0.24^**^ (−0.05)	−0.20^**^ (−0.11)	−0.22^**^ (−0.05)	−0.15^*^ (−0.12)	−0.10 (−0.06)	−0.19^*^ (−0.07)	−0.19^*^ (−0.06)	−0.17^*^ (−0.09)	−0.08 (0.05)
Age 14	−0.09 (−0.10)	−0.11 (−0.11)	−0.05 (−0.08)	0.02 (−0.02)	−0.16^*^ (−0.19^*^)	−0.18^*^ (−0.19^*^)	−0.07 (−0.12)	−0.15 (0.00)	−0.05 (−0.04)	−0.07 (−0.02)	−0.04 (−0.07)	0.08 (0.11)
Age 17	−0.04 (−0.04)	0.01 (−0.01)	−0.03 (−0.02)	−0.02 (−0.01)	−0.11 (−0.14)	−0.11 (−0.11)	−0.06 (−0.11)	−0.08 (−0.03)	−0.11 (−0.23^**^)	−0.11 (−0.26^**^)	−0.02 (−0.18^*^)	−0.13 (−0.09)
**PEQ FATHER REGARD FOR DAUGHTER**												
Age 11	−0.25^**^ (−0.14)	−0.17^*^ (−0.20^**^)	−0.17^*^ (−0.05)	−0.21^**^ (−0.03)	−0.20^**^ (−0.14)	−0.18^*^ (−0.04)	−0.18^*^ (−0.17^*^)	−0.09 (−0.08)	−0.12 (−0.07)	−0.12 (−0.06)	−0.13 (−0.12)	−0.06 (−0.06)
Age 14	−0.04 (0.02)	−0.05 (−0.07)	−0.03 (0.01)	0.01 (0.05)	−0.15 (−0.13)	−0.12 (−0.12)	−0.06 (−0.03)	−0.17^*^ (−0.10)	0.02 (−0.03)	0.04 (−0.07)	−0.00 (−0.08)	0.05 (−0.03)
Age 17	−0.05 (−0.05)	−0.05 (−0.06)	−0.01 (−0.03)	−0.09 (−0.02)	−0.19^*^ (−0.16)	−0.18^*^ (−0.02)	−0.20^*^ (−0.12)	−0.06 (−0.12)	−0.27^**^ (−0.28^**^)	−0.18^*^ (−0.20^*^)	−0.22^**^ (−0.20^*^)	−0.24^**^ (−0.23^**^)
**PEQ DAUGHTER REGARD FOR MOTHER**												
Age 11	−0.21^**^ (−0.21^**^)	−0.14 (−0.05)	−0.17^*^ (−0.18^*^)	−0.23^**^ (−0.23^**^)	−0.20^**^ (−0.11)	−0.10 (−0.05)	−0.13 (−0.02)	−0.23^**^ (−0.16^*^)	−0.24^**^ (−0.03)	−0.06 (0.03)	−0.24^**^ (−0.03)	−0.27^**^ (−0.02)
Age 14	−0.02 (−0.06)	−0.03 (0.03)	0.02 (−0.09)	−0.02 (−0.10)	−0.03 (−0.14)	−0.06 (−0.21^**^)	0.05 (−0.08)	−0.02 (−0.11)	−0.04 (−0.04)	−0.14 (−0.13)	−0.04 (−0.09)	0.13 (0.01)
Age 17	−0.01 (−0.09)	0.04 (−0.01)	0.01 (0.00)	−0.01 (−0.11)	−0.04 (−0.11)	−0.05 (−0.11)	0.04 (−0.05)	−0.11 (−0.10)	0.03 (−0.07)	0.02 (−0.09)	0.08 (−0.06)	−0.04 (0.00)
**PEQ DAUGHTER REGARD FOR FATHER**												
Age 11	−0.26^**^ (−0.22^**^)	−0.14^*^ (−0.09)	−0.19^**^ (−0.15^*^)	−0.22^**^ (−0.25^**^)	−0.19^**^ (−0.10)	−0.12 (−0.04)	−0.14 (−0.01)	−0.18^*^ (−0.16^*^)	−0.10 (−0.02)	−0.03 (0.01)	−0.11 (−0.03)	−0.15 (−0.02)
Age 14	−0.01 (0.00)	−0.02 (−0.06)	−0.03 (−0.04)	0.00 (0.00)	−0.06 (−0.09)	−0.09 (−0.14)	0.01 (0.04)	0.00 (−0.08)	0.08 (−0.06)	0.03 (−0.08)	0.06 (−0.07)	0.13 (0.00)
Age 17	−0.01 (−0.07)	0.01 (−0.06)	−0.01 (0.02)	−0.04 (−0.04)	−0.05 (−0.14)	−0.06 (−0.05)	−0.06 (−0.12)	0.00 (−0.11)	−0.05 (−0.06)	−0.01 (−0.06)	−0.07 (−0.02)	−0.04 (−0.05)
**PEQ MOTHER INVOLVEMENT**												
Age 11	−0.10 (−0.07)	−0.09 (0.03)	−0.06 (−0.13)	−0.15^*^ (−0.06)	−0.07 (−0.09)	−0.03 (−0.05)	−0.02 (−0.04)	−0.12 (−0.01)	−0.10 (−0.02)	0.02 (0.03)	−0.09 (−0.03)	−0.21^**^ (0.03)
Age 14	−0.04 (−0.04)	−0.05 (−0.03)	0.00 (−0.05)	0.01 (−0.02)	−0.06 (−0.07)	−0.09 (−0.17^*^)	0.06 (0.02)	−0.13 (0.04)	−0.04 (0.01)	−0.08 (−0.07)	0.01 (0.01)	−0.03 (0.07)
Age 17	−0.05 (0.00)	−0.01 (−0.01)	−0.02 (0.02)	−0.05 (0.00)	−0.14 (−0.12)	−0.12 (−0.09)	−0.04 (−0.02)	−0.19^*^ (−0.11)	−0.10 (−0.19^*^)	−0.07 (−0.17^*^)	−0.01 (−0.14)	−0.20^**^ (−0.14)
**PEQ FATHER INVOLVEMENT**												
Age 11	−0.20^**^ (−0.14)	−0.12 (−0.03)	−0.16^*^ (−0.15*)	−0.17^*^ (−0.09)	−0.13 (−0.10)	−0.11 (−0.06)	0.08 (−0.02)	−0.12 (−0.07)	−0.06 (−0.02)	0.00 (−0.02)	−0.05 (−0.04)	−0.16^*^ (0.03)
Age 14	−0.08 (−0.05)	−0.06 (−0.11)	−0.08 (−0.04)	−0.01 (−0.02)	−0.19^*^ (−0.11)	−0.18^*^ (−0.15)	−0.08 (−0.01)	−0.21^*^ (−0.13)	−0.04 (0.01)	0.02 (0.04)	−0.03 (−0.05)	−0.04 (0.03)
Age 17	−0.05 (−0.06)	−0.01 (−0.09)	−0.06 (−0.02)	−0.07 (−0.05)	−0.19^*^ (−0.19^*^)	−0.13 (−0.01)	−0.16^*^ (−0.11)	−0.14 (−0.24^**^)	−0.15 (−0.20^*^)	−0.04 (−0.08)	−0.13 (−0.14)	−0.22^**^ (−0.24^**^)

*Total, Minnesota Eating Behaviors (MEBS) Survey Total Score; BD, MEBS Body Dissatisfaction subscale; WP, MEBS Weight Preoccupation subscale; Binge, MEBS Binge Eating subscale; PEQ, Parental Environment Questionnaire*.

* *p < 0.05*,

** *p < 0.01*.

#### Cross-lagged models

**Figure 2:**
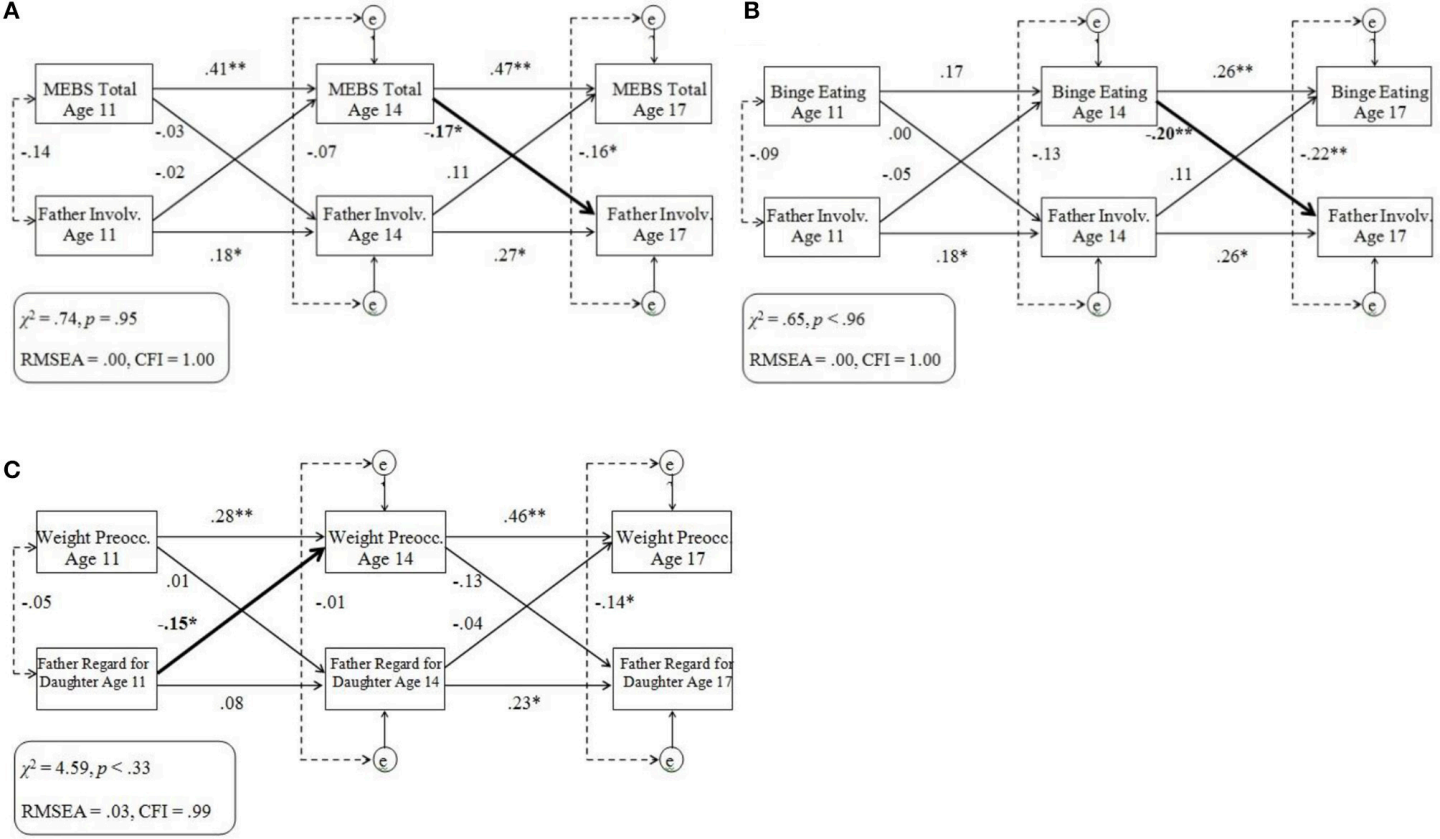
**(A–C)** Cross-lagged models of eating pathology and twin ratings of the parent-child relationship within the full sample. Standardized across-age path coefficients are illustrated by single-headed arrows; within-age correlations are illustrated by dotted lines. Significant cross-lagged associations are denoted in bold. MEBS, Minnesota Eating Behaviors Survey; Involv., Involvement; Weight Preocc., Weight Preoccupation; e, residual variance at ages 14 and 17 years; RMSEA, root mean square error of approximation; CFI, comparative fit index. ^*^p < 0.05, ^**^p < 0.01.

For cross-lagged models that included one or more significant cross-lagged path coefficient, results are displayed in Figures 2A–C. Cross-lagged models without any significant cross-lagged path coefficients are not displayed but can be obtained via contacting the corresponding author. All paths referenced below are labeled in Figure 1.

Almost all cross-lagged models provided adequate fit to the data. Cross-lagged models that included MEBS Body Dissatisfaction scores demonstrated generally poor fit, and a specific model that examined associations between Binge Eating and Father Regard for Daughter demonstrated poor fit. Analyses were re-run on cross-lagged models that included MEBS Body Dissatisfaction, with a longitudinal path added between Body Dissatisfaction at age 11 and 17 years. In all such models, this additional longitudinal path was significant (*p*s < 0.05), improved model fit, and did not change the statistical significance of any cross-lagged path. These findings suggest pre-adolescence Body Dissatisfaction predicts important variance in late-adolescence Body Dissatisfaction.

For both the MEBS and twin PEQ ratings, twin difference scores were more stable during later adolescence (paths b33 and b44) than early adolescence (paths b11 and b22). Regardless, overall low percentage of variance was accounted for across time. Significant negative within-age correlations were generally strongest between MEBS and PEQ difference scores at age 11 (r1) and 17 years (r3).

No significant cross-lagged paths were observed in any models that examined associations between any MEBS difference scores and Daughter Regard for Mother/Father difference scores. However, twin differences in MEBS Total (Figure 2A) and Binge Eating (Figure 2B) at age 14 negatively predicted twin differences in Father Involvement at age 17. In addition, twin differences in Father Regard for Daughter at age 11 negatively predicted twin differences in Weight Preoccupation at age 14 (Figure 2C). The pattern of significant cross-lagged paths was unchanged when cross-lagged models were re-run with BMI regressed out from eating pathology scores.

#### Summary

Cross-lagged longitudinal associations between twin differences in eating pathology and twin perceptions of the parent-daughter relationship were neither strong nor robust. Where significant cross-lagged paths emerged, father-daughter relationship variables more often predicted later eating pathology across early adolescence (ages 11–14), whereas eating pathology more often predicted later father-daughter relationship variables across later adolescence (ages 14–17). In particular, lower twin perceptions of father regard at pre-adolescence predicted greater weight preoccupation at middle adolescence, whereas greater binge eating at middle adolescence predicted lower father involvement at late adolescence. Neither twin ratings of their regard for their mother nor father were associated with eating pathology across time.

### Discordant subsamples

**Figure 3:**
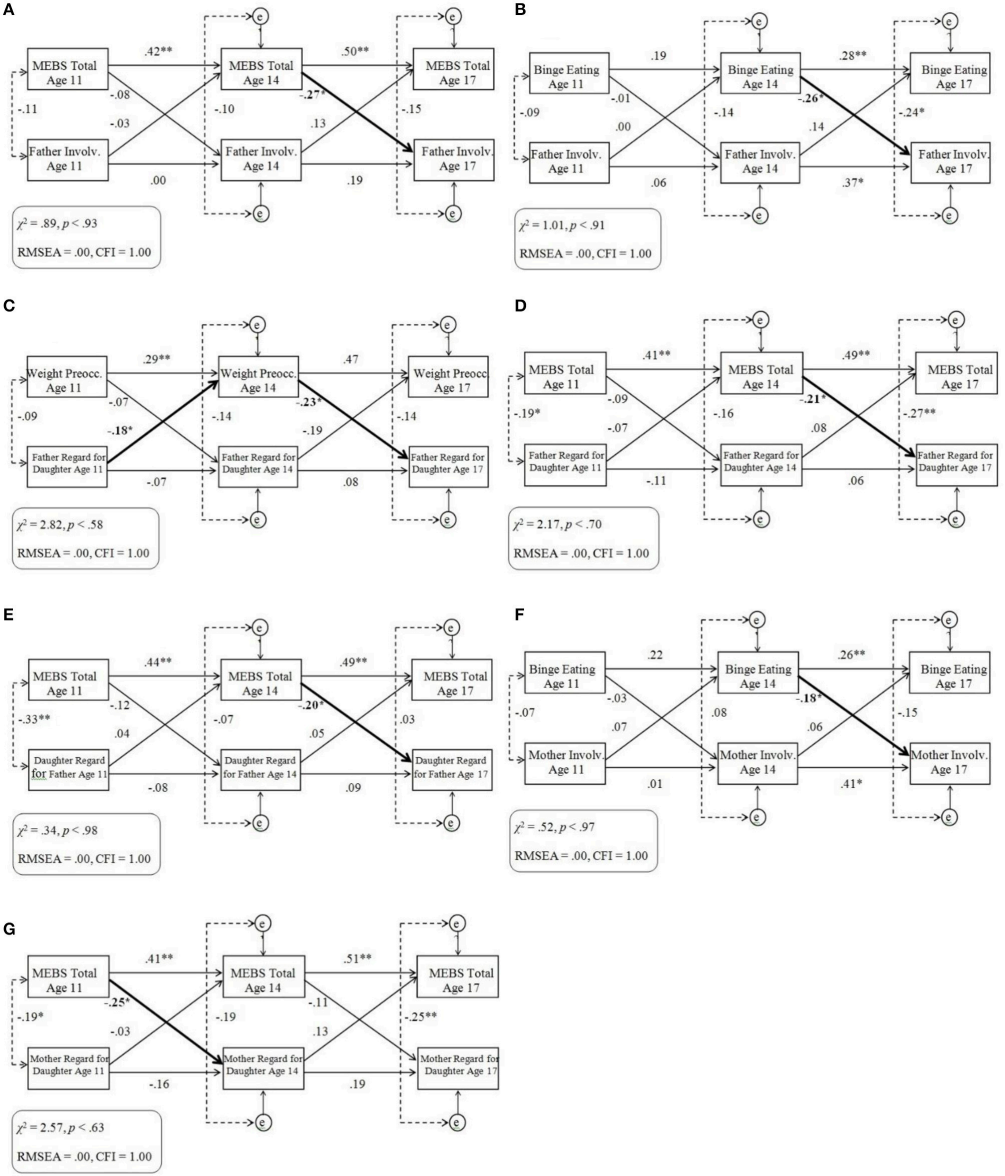
**(A–G)** Cross-lagged models of eating pathology and twin ratings of the parent-child relationship within the discordant sample. Standardized across-age path coefficients are illustrated by single-headed arrows; within-age correlations are illustrated by dotted lines. Significant cross-lagged associations are denoted in bold. MEBS, Minnesota Eating Behaviors Survey; Involv., Involvement; Preocc., Preoccupation; e, residual variance at ages 14 and 17 years; RMSEA, root mean square error of approximation; CFI, comparative fit index. ^*^p < 0.05, ^**^p < 0.01.

For cross-lagged models that included one or more significant cross-lagged path coefficient, results are displayed in Figures 3A–G. Cross-lagged models without any significant cross-lagged path coefficients are not displayed but can be obtained by contacting the corresponding author.

Model fit tended to be adequate, with the exception of models that included the Body Dissatisfaction subscale. As in analyses of the full sample, analyses of models that included the Body Dissatisfaction subscale were re-run with a longitudinal path added between Body Dissatisfaction at age 11 and 17 years, improving model fit and not changing the statistical significance of any cross-lagged path.

All significant cross-lagged paths observed within the full sample remained significant within the discordant subsamples (see Figures 3A–C). In addition, associations tended to be stronger and some additional cross-lagged paths reached the threshold for statistical significance within the discordant subsamples. Specifically, whereas in the full sample twin differences in MEBS Total at age 14 only negatively predicted twin differences in Father Involvement at age 17, in the discordant subsample twin differences in MEBS Total at age 14 additionally negatively predicted twin differences in Father Regard for Daughter (Figure 3D) and Daughter Regard for Father (Figure 3E) at age 17. Likewise, whereas in the full sample twin differences in Binge Eating at age 14 only negatively predicted twin differences in Father Involvement at age 17, in the discordant subsample twin differences in Binge Eating at age 14 additionally negatively predicted twin differences in Mother Involvement at age 17 (Figure 3F). In addition, in the discordant subsample twin differences in MEBS Total at age 11 negatively predicted twin differences in Mother Regard for Daughter at age 14 (Figure 3G) and twin differences in Weight Preoccupation at age 14 negatively predicted twin differences in Father Regard for Daughter at age 17 (Figure 3C).

#### Summary

Associations between twin differences in eating pathology and twin differences in ratings of the parent-daughter relationship were more robust within the discordant subsamples than the full sample. In particular, consistent negative associations were observed between overall levels of eating pathology at middle adolescence and daughters' later perceptions of various aspects of the father-daughter relationship at late adolescence. In addition, negative associations were observed between eating pathology and daughters' later perceptions of mother regard and involvement.

### Maternal perceptions of the mother-daughter relationship

#### Descriptive statistics

Descriptive statistics for maternal PEQ ratings are presented in Table 4. Minimal variability was observed within twin pairs on maternal-rated Mother Regard for Daughter, suggesting this variable may reflect parental characteristics and/or the shared environment more than the non-shared environment. Lack of variability may also reflect reluctance to rate one's regard for each child differentially. Difference scores for maternal ratings of Mother Regard were not included in further analyses due to limited variability.

**Table 4:** Descriptive statistics for maternal ratings of the parent-child relationship.

**Variable**	**Age 11**					**Age 14**					**Age 17**				
	***M***	***SD***	**Min**.	**Max**.	***N***	***M***	***SD***	**Min**.	**Max**.	***N***	***M***	***SD***	**Min**.	**Max**.	***N***
**INDIVIDUAL TWIN SCORES**															
PEQ Mother Regard for Daughter	19.77	0.64	16	20	403	19.70	0.79	15	20	367	19.67	0.79	15	20	330
PEQ Daughter Regard for Mother	28.91	2.5	21	32	403	27.66	2.84	18	32	362	27.59	2.80	15	32	329
PEQ Mother Involvement	44.72	3.39	28	48	403	43.00	3.91	30	48	363	42.34	4.44	28	48	327
**TWIN DIFFERENCE SCORES**															
PEQ Mother Regard for Daughter	0.17	0.48	0	3	200	0.16	0.51	0	4	183	0.21	0.49	0	3	164
PEQ Daughter Regard for Mother	0.67	0.93	0	4	200	0.91	1.06	0	6	180	0.97	0.98	0	4	164
PEQ Mother Involvement	1.03	1.28	0	6	200	1.10	1.64	0	9	181	1.64	1.71	0	8	163

*The absolute values of twin difference scores are presented. PEQ, Parental Environment Questionnaire; min., minimum, max., maximum*.

#### Cross-sectional and longitudinal associations

Fewer statistically significant correlations were observed between MEBS and maternal-rated PEQ scores than observed between MEBS and twin-rated PEQ scores. Effect sizes of significant correlations tended to be small to medium. A minority of correlations between MEBS scores and specific maternal-rated PEQ scores (e.g., Mother Involvement) were stronger when twin difference scores were examined rather than individual twin scores, suggesting associations between variables may at times be masked by genetic and shared environmental effects. Data not shown; detailed correlation matrices can be obtained via contacting the corresponding author.

#### Cross-lagged models

**Figure 4:**
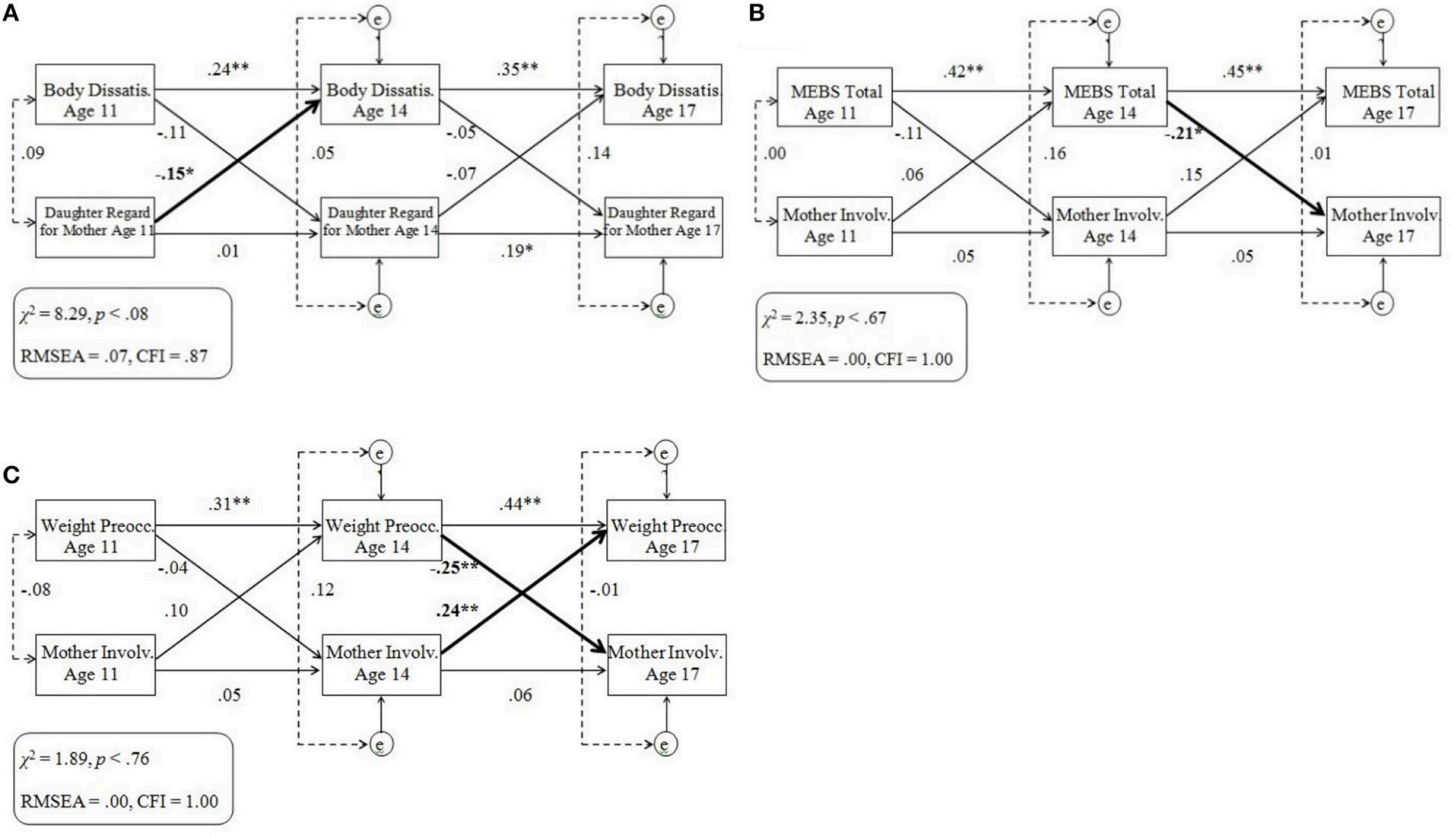
**(A–C)** Cross-lagged models of eating pathology and maternal ratings of the parent-child relationship. Standardized across-age path coefficients are illustrated by single-headed arrows; within-age correlations are illustrated by dotted lines. Significant cross-lagged associations are denoted in bold. MEBS, Minnesota Eating Behaviors Survey; Dissatis., Dissatisfaction; Involv., Involvement; Preocc., Preoccupation; e, residual variance at ages 14 and 17 years; RMSEA, root mean square error of approximation; CFI, comparative fit index. ^*^p < 0.05, ^**^p < 0.01.

For cross-lagged models that included one or more significant cross-lagged path coefficient, results are displayed in Figures 4A–C. Cross-lagged models without any significant cross-lagged path coefficients are not displayed but can be obtained via contacting the corresponding author.

Almost all cross-lagged models that included maternal-rated PEQ scores provided adequate fit to the data, with the exception of models that included the Daughter Regard for Mother (possibly due to lower variability between scores) or the Body Dissatisfaction subscales. As in analyses of twin ratings, analyses of models that included the Body Dissatisfaction subscale were re-run with a longitudinal path added between Body Dissatisfaction at age 11 and 17 years, improving model fit and not changing the statistical significance of any cross-lagged path.

Some significant coefficients were observed on cross-lagged paths. Specifically, twin differences in maternal ratings of Daughter Regard for Mother at age 11 negatively predicted twin differences in Body Dissatisfaction at age 14 (Figure 4A). In addition, twin differences in MEBS Total (Figure 4B) and MEBS Weight Preoccupation (Figure 4C) at age 14 both negatively predicted twin differences in maternal ratings of Mother Involvement at age 17. Twin differences in maternal ratings of Mother Involvement at age 14 predicted twin differences in Weight Preoccupation at age 17 (Figure 4C). Unexpectedly, the direction of association was opposite to other significant associations: the twin who the mother described having greater involvement with later reported greater weight preoccupation. No significant cross-lagged paths were observed in any of the models that examined associations between any maternal-rated PEQ scale and MEBS Binge Eating.

#### Summary

Among cross-lagged models of maternal ratings of the mother-daughter relationship, where significant cross-lagged associations emerged, findings generally followed the pattern that emerged with twin ratings of the parent-daughter relationship in the full and discordant samples: i.e., parent-daughter relationship variables were more likely to predict later eating pathology across early adolescence whereas eating pathology was more likely to predict later parent-daughter relationship variables across later adolescence. However, the specific aspects of eating pathology and the mother-daughter relationship that were most strongly associated varied dependent on whether twins or mothers provided ratings of the mother-daughter relationship.

## Discussion

The current study explored associations between girls' eating pathology and perceptions of the mother-daughter and father-daughter relationship across adolescence. Importantly, this study sought to better understand associations between variables via cross-lagged analyses that examined longitudinal, bidirectional associations and differentiated whether problems in the parent-daughter relationship emerged as a risk factor for, or sequelae of, eating pathology. In addition, this study used an MZ twin differences design that enabled examination of the parent-daughter relationship as a non-shared environmental factor by controlling for influences of genetic and shared environmental factors. Study methodology paralleled methods used by Spanos et al. (2010) in a study of eating pathology and parent-child conflict, and extended analyses to examine different parent-child relationship variables (i.e., parent-child regard and involvement) and address additional limitations of past research.

Overall, longitudinal associations between twin differences in eating pathology and twin differences in the parent-daughter relationship were generally neither strong nor robust. Where longitudinal associations between twin differences in eating pathology and twin differences in the parent-daughter relationship emerged, results indicated some reciprocal associations between symptoms of eating pathology (e.g., binge eating, weight preoccupation) and facets of the parent-daughter relationship (e.g., parent-daughter involvement).

These associations shifted across developmental periods. Specifically, twin differences in the parent-daughter relationship were rarely predictive of subsequent twin differences in eating pathology but, when predictive, this direction of association was only observed across early adolescence. In contrast, twin differences in eating pathology more frequently predicted subsequent twin differences in the parent-daughter relationship, and were particularly evident across later adolescence. These results align with accumulating findings across other studies (e.g., Archibald et al., 1999, 2002), emphasizing that parent-daughter relationships are most predictive of the development of eating pathology during early adolescence. Early adolescence is a developmental period wherein adolescents continue to spend a large portion of time with their parents and rely on them for attachment, security, and emotional needs (De Goede et al., 2009). In comparison, parent-daughter relationships generally do not predict development of eating pathology during later adolescence, a developmental period wherein adolescents spend increasing time with peers and increasingly seek out friends instead of parents for support. Rather, findings suggest eating pathology that develops in middle adolescence may negatively affect the parent-daughter relationship across later adolescence, preceding a decline in the parent-daughter relationship as a possible outcome of eating pathology. These findings generally align with those identified by Spanos et al. (2010), wherein similar methodology and sample uncovered no associations between eating pathology and parent-child conflict during early adolescence but a prospective association between eating pathology (in particular, weight preoccupation) at age 14 years and parent-child conflict at age 17 years. Together, these findings suggest the process of separation and individuation from parents during later adolescence may be accelerated among adolescents with eating pathology, potentially contributing to risk for maintenance of eating pathology and development of other negative outcomes.

### Eating pathology and the father-daughter relationship

Despite the majority of past research related to eating pathology and the parent-child relationship having focused on the mother-child, rather than father-child, relationship, the current study highlights that longitudinal associations between eating pathology and the parent-daughter relationship were more consistently observed with the father-daughter, rather than mother-daughter, relationship. In particular, the majority of significant cross-lagged associations indicated that greater eating pathology predicted development of a poorer father-daughter relationship, particularly during later adolescence. For example, twin differences in both total levels of eating pathology and symptoms of binge eating predicted later twin differences in their perceptions of involvement in the father-daughter relationship. Further, analyses of discordant subsamples highlighted that twin differences in total levels of eating pathology and symptoms of weight preoccupation predicted twin differences in their perceptions of regard within the father-daughter relationship (i.e., daughter's regard for her father and her perceptions of her father's regard for herself). In all these associations, the twin who reported greater eating pathology subsequently rated the father-daughter relationship more poorly than her co-twin.

These longitudinal findings improve our understanding of cross-sectional studies in which women with eating pathology report lower quality of the father-child relationship (e.g., Horesh et al., 2015). In particular, these findings raise questions regarding the way in which fathers respond to daughters who experience eating pathology and suggest the potential importance of efforts to support the father-daughter relationship when an adolescent is experiencing eating pathology. Minimal research has explored paternal perceptions of and responses to daughters' eating pathology. Existing findings suggest fathers may be more likely to use avoidant coping strategies in managing their daughters' eating pathology (Whitney et al., 2005). Research also suggests that effective treatment for eating pathology is associated with a reduction in critical comments between fathers and daughters (Eisler et al., 2000) and paternal perceptions of improved parent-child problem-solving (Ciao et al., 2015).

Only one significant association was observed between eating pathology and twin perceptions of the father-daughter relationship during early adolescence. Specifically, twin differences in perceptions of father's regard for her at pre-adolescence predicted twin differences in weight preoccupation at middle adolescence. That is, the twin who perceived that her father regarded her more poorly later reported greater preoccupation with her weight than her co-twin. Perhaps young girls who perceive they are not viewed favorably by their fathers may become self-conscious of and concerned about their weight after transitioning into puberty. Alternatively, it is possible that perceptions of low regard are a result of weight-related comments and criticism from fathers, which has been demonstrated to predict concerns about weight and weight-control behaviors among adolescents (Neumark-Sztainer et al., 2010). Unfortunately, such variables were not measured in this study.

### Eating pathology and the mother-daughter relationship

No associations were observed between twin differences in eating pathology and twin differences in their perception of the mother-daughter relationship when examined within the full sample, although the following associations emerged within the discordant sample. Across early adolescence, twin differences in total levels of eating pathology later predicted twin differences in their perceptions of their mother's regard; across later adolescence, twin differences in binge eating later predicted twin differences in their perceptions of involvement in the mother-daughter relationship. The direction of both associations indicated the twin who reported greater eating pathology subsequently rated the mother-daughter relationship more poorly than her co-twin. When maternal ratings of the mother-daughter relationship were examined, across later adolescence twin differences in total levels of eating pathology and twin differences in weight preoccupation both later predicted twin differences in maternal perceptions of their involvement in the mother-daughter relationship. The direction of associations indicated that mothers perceived themselves as less involved with the twin who previously reported greater symptoms of eating pathology than her co-twin.

Thus, while associations between eating pathology and ratings of the mother-daughter relationship were less consistently observed than when associations between eating pathology and twin ratings of the father-daughter relationship were examined, the overall trend paralleled that observed among ratings of the father-daughter relationship. That is, development of eating pathology by middle adolescence may present risk to the mother-daughter relationship across later adolescence. In particular, findings suggest eating pathology may be followed by reduced involvement (perceived by both the daughter and mother) in the mother-daughter relationship.

One association also emerged that suggested aspects of the mother-daughter relationship predicted eating pathology, rather than the reverse. Specifically, twin differences in maternal perceptions of daughter regard for her mother at pre-adolescence later predicted twin differences in body dissatisfaction. The twin who was rated by her mother as having lower regard for her mother later reported greater body dissatisfaction than her co-twin. Possibly, a mother who believes one of her daughters does not respect her as highly may respond less warmly and supportively toward that daughter. The daughter may then internalize this relative disapproval by becoming more critical of her own body, leading to greater body dissatisfaction. Relatedly, mothers who use more authoritarian parenting strategies—which have been linked to greater body dissatisfaction and other eating pathology (e.g., Zubatsky et al., 2014)—may be more likely to perceive the adolescent as regarding her less highly due to unreasonable expectations for regard. Notably, this association was not observed when daughters directly rated their regard for their mother. Further research to examine such differences in ratings—including exploring parent-child discordance in perceptions of the parent-child relationship—may be important.

An unexpected association also emerged. Twin differences in maternal perceptions of involvement at middle adolescence later predicted twin differences in weight preoccupation during late adolescence, with the twin who was rated by her mother as having greater involvement in the mother-daughter relationship later reporting greater weight preoccupation than her co-twin. This unexpected association may reflect numerous factors. For example, greater maternal ratings of involvement in the mother-child relationship at age 14 years may reflect a lack of normative parent-child separation and individuation, which has been associated with difficulties in socioemotional adjustment and symptoms of psychopathology, including eating pathology (e.g., Marsden et al., 2002). In addition, it is possible that the PEQ Mother Involvement scale assessed a somewhat different construct at age 14 (e.g., maternal psychological control) as opposed to other ages.

### Discordant subsamples

Associations between twin differences in eating pathology and twin differences in ratings of the parent-daughter relationship were more robust within the discordant subsamples than the full sample. These findings suggest associations between these variables and effects of non-shared environmental processes may be a function of the severity of twin differences in eating pathology and attenuated in unselected, population-based samples. It is also possible more robust associations were observed within the discordant sample due to higher absolute level of eating pathology in the most affected twin. Possibly, associations between eating pathology and the parent-daughter relationship are more likely to emerge when eating pathology passes a threshold of severity.

*Post hoc* analyses compared MEBS Total scale scores between twins who were included within vs. excluded from the discordant sample. Results indicated the twin who reported greater eating pathology within each twin pair included in the discordant sample tended to demonstrate greater eating pathology across time points (*M* = 9.56, *SD* = 4.81 at age 11 years; *M* = 10.67, *SD* = 5.70 at age 14 years; *M* = 10.82, *SD* = 5.59 at age 17 years) than the twin who reported greater eating pathology within each twin pair excluded from the discordant sample (*M* = 4.52, *SD* = 3.67 at age 11 years; *M* = 4.14, *SD* = 4.28 at age 14 years; *M* = 5.09, *SD* = 4.73).

### Implications

As we observed very few significant associations suggesting that a negative parent-daughter relationship was a risk factor for subsequent eating pathology, findings may help reduce stigma and blame that have historically been placed on parents of children with eating disorders. As adolescent treatment-seeking is often initiated by parents, reducing parental stigmatization, and blame for development of their child's eating pathology may be important for increasing treatment-seeking. On the other hand, significant associations that suggested eating pathology was a risk factor for subsequent negative parent-daughter relationships—in particular, twin's negative perception of the father-daughter relationship—are important to consider. These findings suggest development of eating pathology may subsequently challenge parent-child relationships, particularly across later adolescence, highlighting the importance of supporting parent-child relationships among families of adolescents with eating pathology. As later adolescence is a period wherein risk for numerous issues (e.g., substance use, risky sexual behavior, etc.) emerges, addressing negative parent-child relationship factors that may develop secondary to eating pathology may help promote positive adolescent development in general, beyond associations with eating pathology.

For adolescents receiving treatment for eating pathology, these findings suggest interventions may be more successful if they strengthen parent-child relationships, as negative family relationships impact the family's ability to care for those with eating disorders. In particular, younger adolescents presenting to treatment may have parent-child relationship problems that are longer-standing as compared to older adolescents who may have parent-child relationship problems that developed more recently, following expression of eating pathology. It may be important to provide parents with psychoeducation related to eating pathology to help them understand and best respond to symptoms and remain involved and connected in their child's life and recovery. Notably, a primary tenet of family-based therapy for eating disorders, an evidence-based treatment (Couturier et al., 2013), is to mobilize parents as a resource in their adolescent's treatment and reinvigorate parental roles in the family system while showing respect and regard for the adolescent's point of view and experience. It is possible that a mechanism through which family-based therapy promotes change is through preventing and/or addressing the negative parent-child relationship factors that may develop subsequent to eating pathology. Current findings suggest including fathers in treatment may be particularly important.

### Strengths and limitations

Although the MZ twin differences design is touted as the most powerful and direct approach for identifying non-shared environmental influences on development, it also has some limitations. For example, the foundation of the MZ twin differences design lies in the assumption that differential outcomes within pairs can only be the result of non-shared environmental factors, yet the growing field of epigenetics suggests differences within pairs could also be mediated, in part, by the epigenome (Wong et al., 2010). In addition, these findings do not suggest a causal relationship between eating pathology and parent-daughter relationship variables; they identify directions of associations between variables across time. Findings are also limited to understanding *perceptions* of the parent-daughter relationship, which may differ from direct measurements of objective functioning. Only daughters (not sons) were included. Findings only consider limited aspects of the parent-daughter relationship, do not include fathers' perceptions of the father-daughter relationship. In addition, it is possible that analyses of discordant subsamples are underpowered. Further, the magnitude of significant cross-lagged associations was also relatively small, with the earlier variable accounting for only 7% or less of the variance in the later variable. However, recognizing that approximately 3 years passed between each assessment, this variance is not trivial. Several methodologically rigorous longitudinal studies of parenting variables and psychopathology have reported similarly small variance explained (Loukas et al., 2001; Burt et al., 2005).

Despite these limitations, numerous strengths can also be recognized. This study utilized a longitudinal design that considered reciprocal associations between eating pathology and the parent-daughter relationship, including the temporal order of variables. The study design facilitated interpretation of developmental processes associated with narrowly defined age intervals that spanned pre-adolescence to late adolescence. In addition, analyses were genetically informed, enabling isolation of the parent-daughter relationship as a non-shared environmental factor by controlling for both genetics and shared environmental factors. Both daughter's and mother's ratings of the parent-daughter relationship were included and both the mother-daughter and father-daughter relationship were analyzed. In addition, use of a community sample avoided selection biases associated with treatment-seeking samples.

## Conclusion

The current study highlights reciprocal associations between symptoms of eating pathology and perceptions of the parent-daughter relationship that varied dependent on the developmental period examined (i.e., early or later adolescence), the specific relationship being rated (i.e., mother-daughter or father-daughter relationship), and the rater of the relationship (i.e., daughter or mother). Associations between variables were observed when the parent-daughter relationship was isolated as a non-shared environmental factor and influences of genetic and shared environmental factors were controlled. Findings suggested eating pathology experienced by middle adolescence may present risk to the parent-daughter relationship—in particular, the father-daughter relationship—across later adolescence. However, longitudinal associations between eating pathology and the parent-daughter relationship were generally neither strong nor robust and no evidence was identified to support the notion that parents are a primary mechanism underlying risk for eating pathology.

## Ethics statement

This study was carried out in accordance with the recommendations of the American Psychological Association's ethical guidelines, with written informed consent from parents for their own and their children's participation, and children's written informed assent for their participation. The protocol was approved by the University of Minnesota Institutional Review Board.

## Author contributions

LK conceived of the work, conducted data analyses, and drafted the manuscript. KvR supervised and contributed to the conception, design, and execution of the project. WI and SW were involved in data collection and management. KvR, SW, and WI revised the work critically for important intellectual content. All authors contributed to and approved the final manuscript.

### Conflict of interest statement

The authors declare that the research was conducted in the absence of any commercial or financial relationships that could be construed as a potential conflict of interest.

## Footnotes

^1^The MEBS (previously known as the Minnesota Eating Disorder Inventory; M-EDI) was adapted and reproduced for participants by special permission of Psychological Assessment Resources, Inc., 16204 North Florida Avenue, Lutz, Florida 33549, from the Eating Disorder Inventory (collectively, EDI and EDI-2) by Garner, Olmstead, and Polivy (1983). Copyright 1983 by Psychological Assessment Resources, Inc. Further reproduction of the MEBS is prohibited without prior permission from Psychological Assessment Resources, Inc.
